# Synovial fluid monocyte/macrophage subsets and their correlation to patient-reported outcomes in osteoarthritic patients: a cohort study

**DOI:** 10.1186/s13075-018-1798-2

**Published:** 2019-01-18

**Authors:** Alejandro Gómez-Aristizábal, Rajiv Gandhi, Nizar N. Mahomed, K. Wayne Marshall, Sowmya Viswanathan

**Affiliations:** 1 Arthritis Program, University Health Network, Toronto, ON Canada; 2 Krembil Research Institute, University Health Network, Toronto, ON Canada; 3 Cell Therapy Program, University Health Network, Toronto, ON Canada; 4 Division of Orthopaedic Surgery, Toronto Western Hospital, University of Toronto, Toronto, ON Canada; 5 Institute of Biomaterials and Biomedical Engineering, University of Toronto, Toronto, ON Canada; 6 Division of Hematology, Department of Medicine, University of Toronto, Toronto, ON Canada

**Keywords:** Osteoarthritis, Synovial fluid, Monocytes/macrophages, PROMs, Leukocytes

## Abstract

**Background:**

Chronic, low-grade inflammation of the synovium (synovitis) is a hallmark of osteoarthritis (OA), thus understanding of OA immunobiology, mediated by immune effectors, is of importance. Specifically, monocytes/macrophages (MΦs) are known to be abundantly present in OA joints and involved in OA progression. However, different subsets of OA MΦs have not been investigated in detail, especially in terms of their relationship with patient-reported outcome measures (PROMs). We hypothesized that levels of synovial fluid (SF) MΦ subsets are indicative of joint function and quality of life in patients with OA, and can therefore serve as biomarkers and therapeutic targets for OA.

**Methods:**

In this cohort study, synovial fluid leukocytes (SFLs, *N* = 86) and peripheral blood mononuclear cells (*n* = 53) from patients with knee OA were characterized. Soluble MΦ receptors and chemokine (sCD14, sCD163, CCL2, CX3CL1) levels were detected in SF using immunoassays. Linear models, adjusted for sex, age and body mass index, were used to determine associations between SF MΦs and soluble factors with PROMs (*N* = 83). Pearson correlation was calculated to determine correlation between MΦ subsets, T cells and soluble factors.

**Results:**

SF MΦs were the most abundant SFLs. Within these, the double-positive CD14^+^CD16^+^-MΦ subset is enriched in knee OA SF compared to the circulation. Importantly, MΦ subset ratios correlated with PROMs, specially stiffness, function and quality of life. Interestingly, the SF CD14^+^CD16^+^-MΦ subset ratio correlated with SF chemokine (C-C motif) ligand 2 (CCL2) levels but not with levels of sCD163 or sCD14; we found no association between PROMs and either SF CCL2, sCD163, sCD14 or CX3CL1 (which was below detection levels). All SF MΦs displayed high levels of HLA-DR, suggesting an activated phenotype. Correlation between OA SF MΦ subsets and activated CD4^+^ T cell subsets suggests modulation of CD4^+^ T cell activation by MΦs.

**Conclusion:**

SF MΦ subsets are associated with knee OA PROMs and display an activated phenotype, which may lead to modulation of CD4^+^ T cell activation. Knee OA SF MΦ subsets could serve as knee OA function biomarkers and as targets of novel therapeutics.

**Electronic supplementary material:**

The online version of this article (10.1186/s13075-018-1798-2) contains supplementary material, which is available to authorized users.

## Background

Osteoarthritis (OA) is increasingly considered a chronic, low-grade inflammatory disease with involvement of synovial inflammation (synovitis) [1]. Synovitis promotes an inflammatory environment associated with cartilage degradation [2]. In fact, synovitis predicts OA progression and is associated with pain and cartilage degradation [3, 4].

The main immune cells (leukocytes) present in the OA synovium are monocytes/macrophages (defined as a heterogeneous mixture of monocytes and macrophages, MΦs) typically found along the synovial lining layer [5]. MΦs from OA synovium produce inflammatory and degradative mediators [6] and ablation of synovial-resident MΦs reduces OA severity in a murine model [7]. Thus, there is evidence that MΦs contribute to the OA pathogenesis.

Synovitis is often associated with knee fluid effusion [2], suggesting that changes in the synovial fluid (SF) environment happen along with changes in the synovium. Thus, while leukocyte numbers in OA SFs are low (< 2000/mm^3^), these cells may provide information on the inflammatory state of the joint or the patient’s state. Types of leukocytes in knee OA (KOA) SF [8–11] have been quantified: with MΦs in synovial fluid leukocytes (SFLs) showing considerable heterogeneity between studies, with reported frequencies of SFLs of 3–34% [8–11]. Some of these studies have attempted to characterize subpopulations of SF MΦs [10, 11] but the association between SF MΦs and the clinical outcomes has not been studied.

The importance of MΦ-specific factors (i.e. sCD14 and sCD163) [12] and MΦ chemoattractants (CCL2 and CX3CL1) [13–16] have also been investigated in OA SF, and they have been identified as being associated with clinical outcomes [12, 14, 16]. However, the association between these MΦ-specific factors and chemoattractants and specific MΦ subpopulations has not been studied. This is an important area of study as there is growing interest in understanding the effects of MΦ subpopulations on OA pathology.

In circulation, MΦs are classified into three main subtypes based on cell surface antigens: CD14^+^CD16^neg^ (classical), CD14^+^CD16^+^ (intermediate) and CD14^low^CD16^+^ (non-classical). CD14^+^CD16^neg^ MΦs are the most abundant (~ 85%), followed by CD16^+^CD14^low^ (~ 10%) and the double-positive CD14^+^CD16^+^ subset (~ 5%) [17]. The double-positive CD14^+^CD16^+^-MΦ subset frequency increases in systemic inflammation and inflammatory diseases such as rheumatoid arthritis (RA), Crohn’s disease, Eales’ disease and asthma [17–19]. Double-positive CD14^+^CD16^+^-MΦs are considered pro-inflammatory, among the three MΦ subsets, as they preferentially induce T cell activation, have superior production of reactive oxygen species and express the highest levels of human leukocyte antigen–antigen D related (HLA-DR), involved in antigen presentation to CD4^+^ T cells [17, 18, 20].

To date no manuscript has been published on MΦ subsets in OA SF, as defined by CD14 and CD16 markers; however, studies in RA and juvenile idiopathic arthritis SFs show that the double-positive CD14^+^CD16^+^−MΦ subset is increased in SF MΦs when compared to circulating MΦs [21–23]. Similar to circulating MΦs, RA SF double-positive CD14^+^CD16^+^−MΦ express the highest levels of HLA-DR [22]. The specific function of SF MΦ subsets has not been analyzed in any disease; however, the overall RA SF MΦs promote the secretion of inflammatory factors, interleukin-17 and interferon-γ, by T helper cells [21, 24]. This suggests a possible association between higher levels of SF double-positive CD14^+^CD16^+^−MΦs and joint inflammation, at least in RA.

In this cohort study, we investigated the SFL populations and tested whether the double-positive CD14^+^CD16^+^-MΦ subset in SFLs from patients with knee KOA is linked to patient-reported outcome measures (PROMs) and soluble SF MΦ factors. In addition, we determined whether SF MΦs are associated with SF CD4^+^ T cell activation.

## Patients and methods

### Patients with KOA

**Fig. 1:**
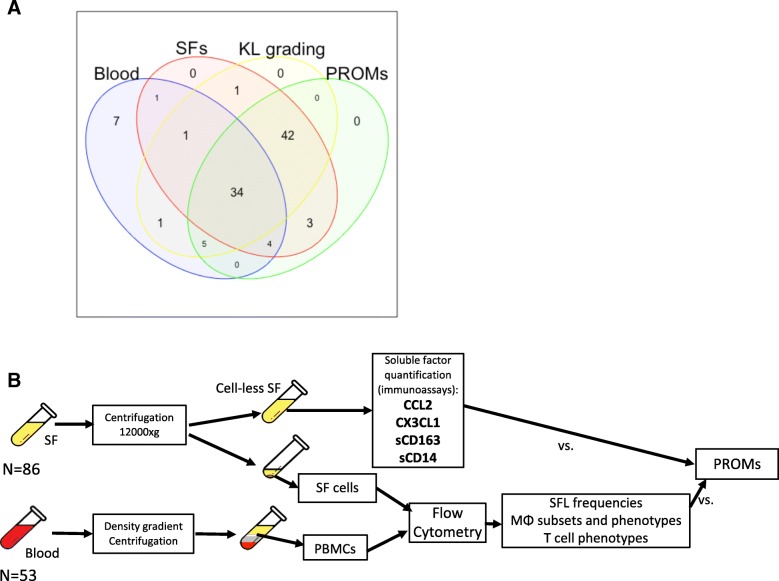
Experimental outline. **a** Venn diagram of samples and data collected: blood, synovial fluids (SFs), Kellgren–Lawrence (KL) grading and patient-reported outcome measures (PROMs). **b** Experimental outline and methods; OA state: i.e. Early (KL I/II) or Late (KL III/IV). PBMCs, peripheral blood mononuclear cells; MΦ, monocytes/macrophages; SF, synovial fluid; CCL2, chemokine (C-C motif) ligand 2

Patients diagnosed with KOA (n = 86) donated their SF at the time of clinical intervention (intra-articular injection, arthroscopy or arthroplasty). Blood from 53 patients with KOA was also acquired, 40 which were also SF donors (Fig. 1a). Patients provided written informed consent to participate in this study, approved by the University Health Network Research Ethics Board (Protocol ID 14–7483-AE). All enrolled patients met the American College of Rheumatology criteria for symptomatic KOA [25]. Exclusion criteria included history of inflammatory arthritis, intra-articular corticosteroid injection (within 3 weeks of surgery) and blood dyscrasias.

Radiographs from 78 of the 86 patients were available for Kellgren–Lawrence (KL) grading. Radiographs were scored by an experienced surgeon for KL grade. Early-stage KOA was defined as KL grades I/II and late-stage as grades III/IV.

### Assessment of PROMs

Functional and symptomatic assessment was evaluated using PROMs. Briefly, Knee Injury and Osteoarthritis Injury Score (KOOS) questionnaires [26] were filled out prior to clinical intervention (up to 3 months before the intervention, *N* = 83; 3 patients who donated SF did not fill out questionnaires). Symptoms, pain, activity of daily living (ADL), sports and quality of life (QOL) KOOS subscales, and Western Ontario and McMaster Universities Osteoarthritis Index (WOMAC) pain, stiffness and function were determined from the questionnaire responses [26]. Mean KOOS and WOMAC were determined by averaging all KOOS and WOMAC subscales, respectively. All KOOS and WOMAC scores are presented in a scale from 0 to 100, where 0 is the worst outcome.

### SF and peripheral blood mononuclear cell (PBMC) isolation

SFs and blood were acquired at the time of the intervention, stored at 4 °C and processed within 24 h of acquisition (Fig. 1b). Further details are in Additional file 1: Supplementary Methods.

### Measurement of cell populations in SFs and PBMCs

MΦs and their subpopulations were identified based on Abeles et al., 2012 [27] (Additional file 1: Figure S1), using FlowJo v10. Neutrophils were identified as CD16^+^HLA-DR^neg^ with high granularity while natural killer (NK) cells are those with low granularity. MΦs were CD14^+^ and/or CD16^+^ while expressing HLA-DR at different levels. Additional details on cell labelling are presented in Additional file 1: Supplementary Methods.

### Measurements of chemotactic factors and soluble receptors

SF supernatants were digested with hyaluronidase, to reduce viscosity, using a 1:1 SF: hyaluronidase solution in RPMI at 500 U/ml for 1 h at room temperature with circular shaking (600 rpm) (Fig. 1b). The chemokine (C-C motif) ligand 2 and chemokine (C-X3-C motif) ligand 1 (CX3CL1) were detected using ProcartaPlex simplex assay as per manufacturer’s recommendations (ThermoFisher). The intra-assay and inter-assay CV was 7.5% and 14.2%, respectively, for CCL2, and the intra-assay and inter-assay CV for CX3CL1 was 10%. A dilution of 1:10 was used (with kit=specific buffer) to reduce interference of SF matrix on the assay. sCD14 (x 1000 dilution) and sCD163 (x 400 dilution) were detected using ELISA kits and diluted with kit-specific solutions (ThermoFisher); the inter-assay and the intra-assay CV is < 12% and < 10%, respectively.

### Statistical analysis

Descriptive statistics were obtained (Additional file 1: Tables S1 and S2). The Wilcoxon sign-rank (paired) test was used to compare blood and SF samples and to compare between populations within the same sample. Bootstrapping was used to perform two-sided tests for estimation of Pearson correlation coefficients and linear modeling on samples to analyze the empirical distribution [28] and thus overcome the need to assume normality in either the samples or residuals; however the *t* values were confirmed as normal by histogram and normal quantile plot after bootstrap; 10,000 bootstrapping samples were taken and *P* values calculated by evaluating the hypothesis of P(*r* = 0|H0) using bootstrapped estimates [29] and the adjusted *P* value was acquired by adjusting for multiple comparisons (for each set of comparisons) as per the method of Li and Ji [30]: briefly, the correlation matrix of the parameters evaluated for each set of comparisons (e.g. cell population frequencies, PROMs) was calculated, followed by estimating the effective number of comparisons (Meff) from the eigen values (λ) of the correlation matrix as per the equation Li and Ji [30] proposed; the adjusted *p* value was then calculated as per the equation:

1-(1-*p* value)^Meff^

Confidence intervals (CI) were calculated using the adjusted bootstrap percentile. For linear models, the effect estimates (β), which indicate the slope for the given variable, is presented along CIs. Our linear models are adequately powered for a multiple R square of 0.133 (using four predictors) or 0.154 (using five predictors), alpha = 0.05 and power = 0.8.

## Results

The median age and body mass index (BMI) of our cohort (*n* = 83, used for linear modeling) was 62 years and 29.75 kg/m^2^, respectively: 60% of patients were female; 49% underwent arthroplasty. Within the patients graded by KL (*N* = 76), 31.6% were in early-stage KOA (KLI/II). Median values for KOOS and WOMAC subscales were 44.4–50 for WOMAC and KOOS pain, KOOS ADL, WOMAC function and WOMAC stiffness. The medians for KOOS QOL and KOOS sports were 18.8 and 15, respectively (Additional file 1: Table S1). Use of pain medications is tabulated in Additional file 1: Table S2; and no effects on MΦ subset frequencies in total SF MΦs was seen from use of pain medications (Additional file 1: Figure S2).

### Leukocytes in KOA SF

**Fig. 2:**
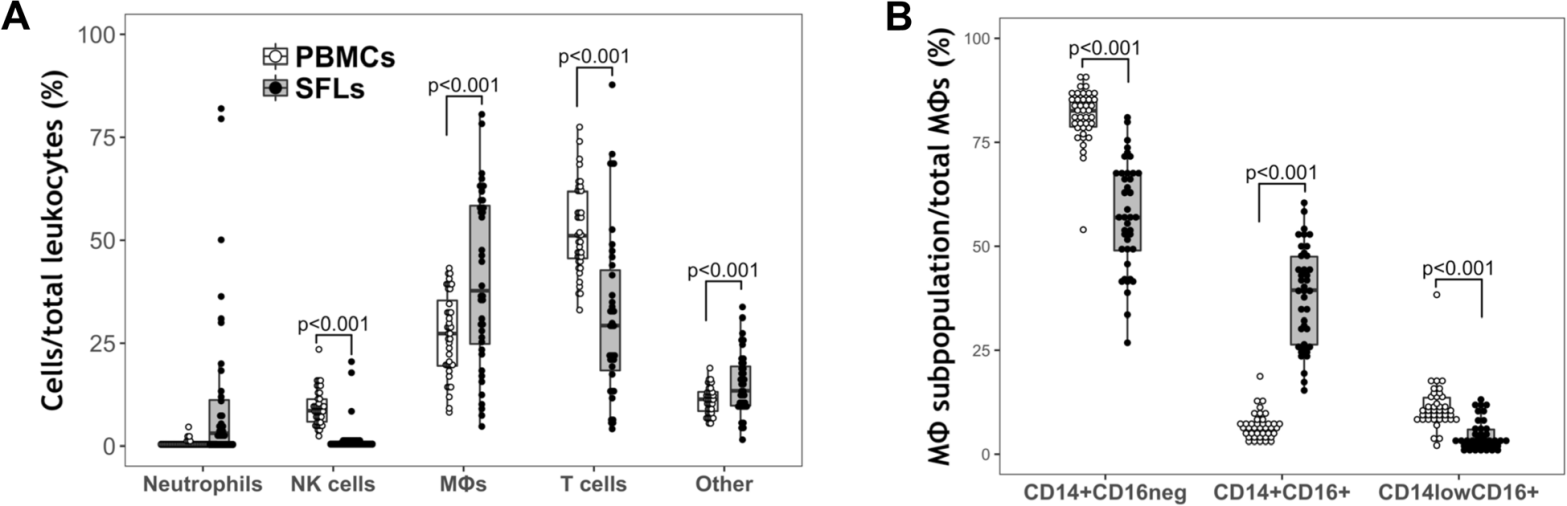
Knee osteoarthritis (KOA) synovial fluid (SF) monocytes/macrophages (MΦs) are the most abundant SF leukocytes (SFLs) and have different prevalence to those in the circulation. **a** Frequency of SFL and peripheral blood mononuclear cell (PBMC) (*N* = 40) populations. No statistical analysis is shown for neutrophils. **b** MΦ subset ratios, in PBMC and SF MΦs (*N* = 40). **a** and **b** Wilcoxon signed test (paired) was used. *P* values shown are corrected for multiple comparisons. Box plots indicate median with interquartile range for box and Tukey-style whiskers. Lines between boxplots indicate significant differences, with adjusted *p* value shown on top. White dots with white box represent data from PBMCs; black dots with gray box represent data from SFLs. NK, natural killer

The main leukocyte populations in KOA SFLs were MΦs (median = 36.5%), followed by T cells (median = 31.1%; with *p* < 0.001 for T cells vs. MΦs, Fig. 2a). Neutrophils were not abundant in KOA SFs, with a median SFL frequency of 2.95%; only 12% (*N* = 75) of patients had > 20% neutrophils in their SFLs.

Since most KOA SFLs (> 97.0%) are mononuclear, PBMCs (*N* = 40) were used as a control to benchmark SFLs. Relative to circulation, MΦs were enriched in KOA SFLs while T and NK cells were diminished (Fig. 2a).

Further analysis of MΦ subsets using CD14 and CD16 markers, typically used for classifying blood monocytes [17] (Additional file 1: Figure S1), showed that KOA SF MΦs are selectively enriched for the double-positive CD14^+^CD16^+^ subset (39.4% vs. 6.3% in circulation); both CD14^+^CD16^neg^ (57.00%) and CD14^low^CD16^+^ (3.34%) subsets were reduced in frequency compared to circulating MΦs in patients with KOA (82.5% and 10.7% for CD14^+^CD16^neg^ and CD14^low^CD16^+^, respectively) (Fig. 2b). Interestingly, some MΦs in OA SF expressed the mature macrophage marker 25F9 [31]. This marker was not expressed on circulating MΦs, indicating the lack of mature macrophages. There was higher frequency of 25F9 expression on SF CD14^low^CD16^+^-MΦs (30.9%) than on SF double-positive CD14^+^CD16^+^-MΦs (17.3%), and only a few 25F9+ MΦs in the CD14^+^CD16^neg^ subset (5.43%) (Additional file 1: Figure S3C).

These data indicate that MΦs are the most abundant SFLs and are enriched for the putative pro-inflammatory double-positive CD14^+^CD16^+^-MΦ subset.

### MΦ subset ratios correlate with PROMs

Linear modeling, adjusted for sex, BMI and age, known confounders of KOA severity [32–34], showed that both CD14^+^CD16^neg^-MΦs/total SF MΦs and double-positive CD14^+^CD16^+^-MΦs/total SF MΦs are significant predictors of mean KOOS and WOMAC scores (Table 1), with a 1% increase in either of these MΦ ratios leading to a 0.38–0.47-point change in mean KOOS or WOMAC scores. These two MΦ subsets were closely correlated, as they comprised 96.4% of all SF MΦs; an increase in one subset would typically come at the expense of the other subset. With this in mind, CD14^+^CD16^neg^-MΦs/total SF MΦs was a significant predictor of all subscales except KOOS-symptoms, WOMAC-pain and KOOS-pain subscales. The double-positive CD14^+^CD16^+^-MΦs/total SF MΦs displayed more specificity for predicting KOOS-QOL and WOMAC-stiffness than CD14^+^CD16^neg^-MΦs/total SF MΦs (Table 1). A 1% change in the double-positive CD14^+^CD16^+^-MΦs/total SF MΦs ratio affects KOOS-QOL and WOMAC-stiffness scores by 0.57 and 0.58 points, respectively, vs. 0.45 for both subscales from a 1% change in the CD14^+^CD16^neg^-MΦs/total SF MΦs ratio.

**Table 1:** Effect size of ratio of MΦ subsets in total SF MΦs with patient reported outcome measures (PROMs)

	CD14^+^CD16^neg^-MΦs/total SF MΦs (%)	CD14^+^CD16^+^-MΦs/total SF MΦs (%)	CD14^low^CD16^+^-MΦs/total SF MΦs (%)
	β (95% CI), adjusted *p*	β (95% CI), adjusted *p*	β (95% CI), adjusted *p*
Overall			
mean KOOS	**0.379 (0.179, 0.610),** ***p*** **= 0.006**	**−0.376 (− 0.642, − 0.132),** ***p*** **= 0.025**	−0.761 (− 1.601, − 0.030), *p* = 0.115
mean KOOS^a^	**0.380 (0.193, 0.613), p = 0.003**	**− 0.415 (− 0.682, − 0.178),** ***p*** **= 0.005**	−0.545 (− 1.191, 0.048), *p* = 0.247
mean WOMAC	**0.431 (0.191, 0.710),** ***p*** **= 0.010**	**−0.459 (− 0.769, − 0.170),** ***p*** **= 0.018**	−0.734 (− 1.738, 0.059), *p* = 0.224
mean WOMAC^a^	**0.380 (0.193, 0.613),** ***p*** **= 0.003**	**−0.473 (− 0.800, − 0.159),** ***p*** **= 0.023**	−0.500 (− 1.335, 0.138), *p* = 0.463
KOOS			
SYMPTOMS	0.187 (−0.055, 0.453), *p* = 0.459	−0.177 (− 0.491, 0.114), *p* = 0.667	−0.372 (− 1.227, 0.479), *p* = 0.869
SYMPTOMS^a^	0.188 (− 0.040, 0.471), *p* = 0.437	− 0.228 (− 0.539, 0.045), *p* = 0.389	−0.153 (− 0.904, 0.573), *p* = 0.988
PAIN	0.299 (0.066, 0.556), *p* = 0.069	−0.295 (− 0.594, 0.013), *p* = 0.215	−0.633 (− 1.526, 0.069), *p* = 0.271
PAIN^a^	0.288 (0.023, 0.579), *p* = 0.156	−0.308 (− 0.642, 0.016), *p* = 0.244	−0.450 (− 1.195, 0.202), *p* = 0.554
ADL	**0.477 (0.202, 0.796),** ***p*** **= 0.013**	−0.458 (− 0.816, − 0.117), *p* = 0.054	− 1.094 (− 2.214, − 0.169), *p* = 0.052
ADL^a^	**0.482 (0.201, 0.797),** ***p*** **= 0.010**	**−0.492 (− 0.845, − 0.137),** ***p*** **= 0.038**	−0.861 (− 1.788, − 0.127), *p* = 0.092
QOL	**0.431 (0.170, 0.702), p = 0.010**	**− 0.497 (− 0.792, − 0.215),** ***p*** **= 0.008**	−0.463 (− 1.508, 0.432), *p* = 0.813
QOL^a^	**0.453 (0.253, 0.667),** ***p*** **= 0.002**	**−0.571 (− 0.826, − 0.357), q < 0.001**	−0.231 (− 0.954, 0.459), *p* = 0.932
SPORTS	**0.502 (0.223, 0.869),** ***p*** **= 0.009**	−0.454 (− 0.901, − 0.101), *p* = 0.104	**−1.244 (− 2.164, − 0.512), p = 0.005**
SPORTS^a^	**0.491 (0.213, 0.865),** ***p*** **= 0.014**	−0.475 (− 0.925, − 0.122), *p* = 0.071	**−1.033 (− 1.839, − 0.347),** ***p*** **= 0.031**
WOMAC			
PAIN	0.322 (0.084, 0.608), *p* = 0.059	−0.327 (− 0.659, − 0.035), *p* = 0.137	−0.655 (− 1.616, 0.116), *p* = 0.330
PAIN^a^	0.312 (0.040, 0.616), *p* = 0.122	−0.347 (− 0.711, − 0.016), *p* = 0.167	−0.378 (− 1.203, 0.318), *p* = 0.753
FUNCTION	**0.477 (0.202, 0.795), p = 0.013**	−0.458 (− 0.816, − 0.117), p = 0.054	−1.093 (− 2.213, − 0.168), *p* = 0.053
FUNCTION^a^	**0.482 (0.201, 0.797), p = 0.010**	**−0.492 (− 0.845, − 0.137),** ***p*** **= 0.037**	−0.860 (− 1.788, − 0.127), p = 0.092
STIFFNESS	**0.479 (0.194, 0.788), p = 0.010**	**− 0.592 (− 0.922, − 0.274),** ***p*** **= 0.007**	−0.454 (− 1.517, 0.413), *p* = 0.815
STIFFNESS^a^	**0.453 (0.159, 0.777),** ***p*** **= 0.019**	**−0.580 (− 0.924, − 0.247), p = 0.010**	−0.262 (− 1.217, 0.450), *p* = 0.951

All correlations adjusted for sex, body mass index and age; *N* = 83. Values in boldface show results with significant effect estimates (β)

*MΦ* monocytes/macrophages, *SF* synovial fluid, *SFLs* synovial fluid leukocytes, *KOOS* Knee Injury and Osteoarthritis Injury Score, *WOMAC* Western Ontario and McMaster Universities Osteoarthritis Index, *ADL* activities of daily living, *QOL* quality of life

^a^Additionally adjusted for osteoarthritis state (i.e. early, Kellgren–Lawrence (KL) grade I/II, *n* = 24 or late, KL grade III/IV, *n* = 52); *N* = 76

Interestingly, the third subset, CD14^low^CD16^+^-MΦs/total SF MΦs was not associated with mean KOOS or WOMAC but was a good predictor of the KOOS-sports subscale. These data reveal that MΦ subset ratios can indicate different aspects of functional outcomes in patients with KOA.

### MΦ-shed receptors are not associated with PROMs

Levels of sCD14 and sCD163, shed from MΦs, have previously been associated with structural changes in KOA and in the case of sCD14, with KOA symptoms [12]. We investigated whether these shed receptors correlate with specific MΦ subsets; levels of sCD14 and sCD163 in KOA SF correlated with each other (*r* = 0.630 *p* < 0.001), as previously shown [12] (Additional file 1: Figure S5). In our hands, however, neither sCD163 nor sCD14 were predictors of any PROMs upon adjustment for sex, age and BMI, or additional adjustment for OA stage (Additional file 1: Table S5). There was significant correlation between levels of SF sCD14 and double-positive CD14^+^CD16^+^-MΦs/SFLs, reflective of correlation between sCD14 and the overall frequency of MΦs in SFLs (Table 2, Additional file 1: Figure S7).

**Table 2:** Correlation between MΦ subsets and SF sCD163 and sCD14

	sCD163 (ng/ml)		sCD14 (ug/ml)	
	Pearson’s *r*, CI	Adjusted p	Pearson’s *r*, CI	Adjusted p
CD14^+^CD16^+^-MΦs/total SF MΦs	−0.007 (− 0.263, 0.232)	1.000	−0.044 (− 0.306, 0.226)	0.996
CD14^+^CD16^neg^-MΦs /total SF MΦs	0.009 (− 0.231, 0.250)	1.000	0.073 (− 0.185, 0.318)	0.967
CD14^low^CD16^+^-MΦs /total SF MΦs	− 0.008 (− 0.186, 0.270)	1.000	−0.110 (− 0.300, 0.091)	0.708
Total MΦs/total SFLs	− 0.147 (− 0.334, 0.074)	0.695	**−0.304 (− 0.491, − 0.073)**	**0.028**
CD14^+^CD16^+^-MΦs/total SFLs	−0.131 (− 0.329, 0.097)	0.654	**−0.294 (− 0.482, − 0.054)**	**0.043**
CD14^+^CD16^neg^-MΦs/total SFLs	−0.134 (− 0.328, 0.090)	0.614	−0.225 (− 0.428, 0.000)	0.165
CD14^low^CD16^+^-MΦs/total SFLs	− 0.053 (− 0.218, 0.226)	0.969	−0.223 (− 0.390, − 0.018)	0.111

*N* = 81. Values in boldface show results with significant effect estimates (β)

*MΦ* monocytes/macrophages, *SF* synovial fluid, *SFL,* synovial fluid leukocytes

These data indicate that there is no association between SF MΦ subset ratios (in total MΦs) and the levels of shed receptors, however the correlation between sCD14 and the overall frequency of MΦs and the double-positive CD14^+^CD16^+^ subset in SFLs reveals associations between MΦ frequencies to previously investigated biomarkers of OA severity.

### SF KOA CD14^+^CD16^+^ MΦ subset is associated with SF CCL2 levels

**Fig. 3:**
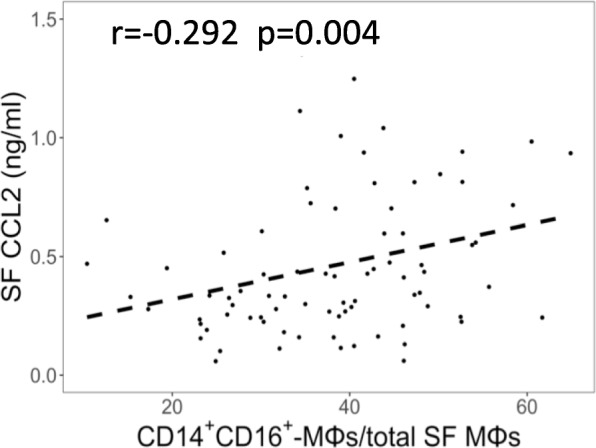
Pro-inflammatory monocytes/macrophages (MΦ) subset correlate with synovial fluid (SF) chemokine (C-C motif) ligand 2 (CCL2) levels. Positive correlation between SF CCL2 levels and CD14^ +^ CD16^ + ^−MΦs (to total SF MΦs) ratio, *N* = 81. Pearson *r* and adjusted *p* value. Dashed line indicates regression resulting from general linear modeling approximation

We investigated whether the level of CCL2, an important MΦ chemoattractant [17] and agonist of CCR2, is associated with the ratios of MΦ subsets from OA SF. CCL2 was present at 343.2 pg/ml (median) in KOA SF and its levels correlated with the ratio of double-positive CD14^+^CD16^+^-MΦs/total SF MΦs (adjusted *p* = 0.004, Fig. 3). There was no significant correlation between CCL2 and the other MΦ subsets (Additional file 1: Table S6) and CCL2 was not associated with PROMs either (Additional file 1: Table S7). Interestingly, SF double-positive CD14^+^CD16^+^-MΦs had the highest frequency of CCR2^+^ cells (89.1%, Additional file 1: Figure S3A) and expressed the highest levels of CCR2 per cell among SF MΦs; conversely circulating CD14^+^CD16^neg^-MΦs expressed the highest levels of CCR2 per cell (Additional file 1: Figure S3B).

An alternative modulation of the SF MΦ ratio could be through CX3CL1, a chemokine that signals to both double-positive CD14^+^CD16^+^ and CD14^low^CD16^+^ MΦ subsets [20]. However, we were unable to detect this chemokine in KOA SFs (lowest detection limit 11.7 pg/ml).

### KOA SF MΦs are in an activated state

**Fig. 4:**
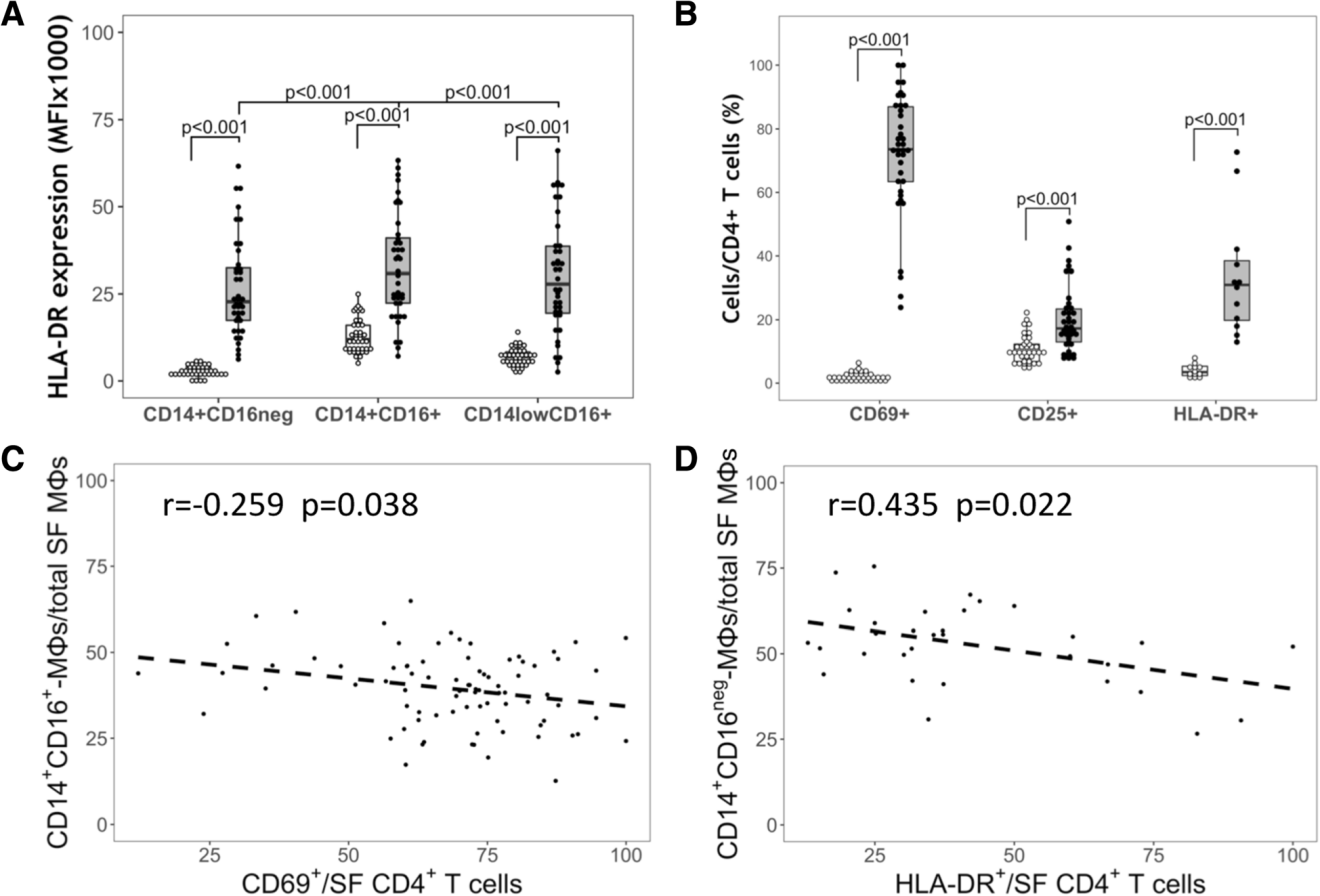
Knee osteoarthritis (KOA) synovial fluid (SF) monocytes/macrophages (MΦ) subsets and CD4+ T cells display an activated phenotype and correlate with each other. **a** Levels of HLA-DR expression in MΦs: mean fluorescence intensity (MFI) on peripheral blood mononuclear cell (PBMC) and SF MΦ subpopulations (SF leukocytes (SFLs), *N* = 40). **b** Frequency of activated cells in CD4^+^ T cells: CD69 + (early activation; *N* = 37), CD25+ (intermediate activation, SFLs, *N* = 37), HLA-DR+ (late activation, SFLs, *N* = 15). **c** Negative correlation between CD14^+^CD16^+^-MΦs (to total SF MΦs) ratio and the frequency of early activated (CD69^+^) CD4^+^ T cells; *N* = 82. **d** Negative correlation between CD14^+^CD16^neg^-MΦs (to total SF MΦs) ratio and the frequency of late activated (HLA-DR^+^) CD4^+^ T cells; *N* = 32. **a** and **b** Median with interquartile range for box and Tukey-style whiskers. Lines between boxplots indicate significant differences with adjusted *p* value on top. White dots with white box represent data from PBMCs; black dots with gray box represent data from SFLs. **c** and **d** Pearson correlation coefficient (*r*) and adjusted *p* value shown

HLA-DR is one of the major histocompatibility complexes and is used by pro-inflammatory MΦs to present antigens to CD4^+^ T cells, leading to their activation [35]. MΦs in KOA SFs have higher expression of HLA-DR compared to those in circulation (*N* = 40, Fig. 4a), indicating they are in an activated state [36]. HLA-DR expression (on a per cell basis) is the highest on SF double-positive CD14^+^CD16^+^-MΦs (Fig. 4a), supportive of a pro-inflammatory function in this subset.

Further evidence of pro-inflammatory MΦ activation was investigated indirectly by determining the activation of SF CD4^+^ T cells and their associations with MΦ subsets. Thus, using markers of early (CD69), intermediate (CD25) and late T cell activation (HLA-DR), we determined that 71.70% of KOA SF CD4^+^ T cells are CD69^+^, 19.94% are CD25^+^ and 36.35% are HLA-DR^+^, indicating a spectrum of activated T cells in KOA SFs (Fig. 4b). We also found negative correlation between the ratio of double-positive CD14^+^CD16^+^-MΦ/total SF MΦs vs. CD69^+^/CD4^+^ T cells (Fig. 4c), and the ratio of CD14^+^CD16^neg^-MΦ/total SF MΦs vs. HLA-DR^+^/CD4^+^ T cells (Fig. 4d). Taken together, KOA SF MΦ subsets were associated with the degree of SF CD4^+^ T cell activation, indicating further evidence of an activated MΦ state.

## Discussion

To our knowledge, this is the first study to investigate the ratios of KOA SF MΦ subsets and their association with patient-reported outcomes and SF soluble factors. We provide evidence that (1) SF MΦs in KOA have a high prevalence of putative pro-inflammatory subtypes; (2) SF MΦ subsets are associated with PROMs; and (3) SF MΦs are activated and associated with SF CD4^+^ T cell activation, potentially promoting a more adaptive immune response.

Our data on SFL populations are comparable to other reports [10, 11]. However, they also differ from frequencies reported by Penatti et al. [9] or Jónasdóttir et al. [8]; the differences observed in those two reports can be attributed to the differences in SFL isolation from the SF: both reported using centrifugation forces lower than 2000×g, which based on our experience, with the high viscosity of most KOA SF samples, does not allow for full cell pelleting, with many cells remaining in suspension.

Interestingly, our profile of SF MΦs is similar to the SF MΦs profile in RA [21, 22]; although the RA SFLs are considerably more abundant than OA’s, consistent with marked inflammation of the synovium in RA [21]. Similar to our reports, the double-positive CD14^+^CD16^+^-MΦ subset is also increased in RA SF MΦs when compared to circulating MΦs [21].

We showed, for the first time, that SF MΦ subsets are partially explanatory of KOOS and WOMAC scores. Both CD14^+^CD16^neg^ and CD14^+^CD16^+^-MΦ subsets were good predictors of mean KOOS and WOMAC scores; we think that since an increase in one subset (i.e. double-positive CD14^+^CD16^+^-MΦs) typically comes at the expense of the other subset (i.e. CD14^ +^ CD16^neg^-MΦs), association between PROMs and the CD14^+^CD16^neg^-MΦs/total SF MΦs ratio is a result of an active increase in the double-positive CD14^+^CD16^+^-MΦs. We thus hypothesize that double-positive CD14^+^CD16^+^-MΦs may be the actual effectors that lead to changes in patients’ function and quality of life as they had higher effect estimates (β) for WOMAC stiffness and KOOS quality of life. Importantly, none of the associations between PROMs and MΦ subset ratios were dependent on radiographic OA stage. This supports previous results showing correlation between synovitis scores and pain, but not radiographic OA grading [3]. However, we did observe higher effect estimates in subcohorts with early stage vs. late stage KOA.

Our data on sCD14 differed from parts of the data reported by Daghestani et al. [12]. We show that sCD14 is correlated with some KOA PROMs (KOOS and WOMAC vs. First National Health and Nutrition Examination Survey criterion [12]), but only when *not* adjusted for confounders. The lack of correlation with adjustments may be due to differences in cohorts as previously noted [12] and the different criteria used to evaluate PROMs. Similarly, we did not find any significant association between CCL2 levels and PROMs and we failed to detect any CX3CL1 in the SFs from our cohort. This differs from the report from Li and Jian [16], which showed a significant association between SF CCL2 levels and WOMAC subscales; interestingly their median SF CCL2 of 44.8 ng/ml was at least two orders of magnitude greater, indicating that cohorts are significantly different. Similarly, the measurable levels of CX3CL1 by Huo et al. [14] in OA SF and its association with PROMs may be related to the cohort chosen.

We speculate that the measurable levels of the chemokine CCL2 present in KOA SFs indicate a mechanism of SF MΦ recruitment from circulation, which is corroborated by expression of CCR2 on most SF MΦs. Expression of CCR2 (exclusive to monocytes rather than macrophages [31]) suggests presence of recruited circulating monocytes into KOA SFs rather than presence of resident macrophages shed from the synovial lining. Murine studies also show that inhibition of CCL2/CCR2 signaling leads to decreased synovial MΦs [37–39], indicating less MΦ recruitment, and concomitant reduction in pain [37, 38] and cartilage degradation [39]. In addition, the expression of the mature macrophage marker, 25F9 [31] at higher proportions on SF CD14^low^CD16^+^-MΦs (30.9%) than on SF double-positive CD14^+^CD16^+^-MΦs (17.3%) suggests that SF MΦs are likely recruited from circulation as CD14^+^CD16^neg^-monocytes, subsequently maturing into double-positive CD14^+^CD16^+^-MΦs, and finally into CD14^low^CD16^+^-MΦs [40], based on joint environmental cues.

The activation state of KOA SF MΦs has previously been reported for SF MΦs in RA where SF MΦs regulate CD4^+^ T cell responses [21]. Our data show that KOA SF MΦ subsets are associated with the levels of early and late activation in CD4^+^ T cells, suggesting that KOA SF MΦ subsets may functionally serve as possible modulators of SF CD4^+^ T cell activation. Specifically, we hypothesize that SF double-positive CD14^+^CD16^+^-MΦs may enable transition from early-activated to late-activated CD4^+^ T cells in the KOA joint. This hypothesis is based on the negative correlation between double-positive CD14^+^CD16^+^-MΦs/total SF MΦs and early-activated CD4^+^ T cells (CD69^+^), and the positive correlation between CD16^+^-MΦs (89.5% being double-positive CD14^+^CD16^+^- MΦs)/total SF MΦs and late-activated CD4^+^ T cells (HLA-DR^+^). Future studies may test this hypothesis, but due to the low numbers of KOA SFLs, the tests required are presently unfeasible.

While we did not focus our study on determining whether SFLs reflect synovium leukocytes, preliminary data from our laboratory (8 synovium samples vs 75 SFs) show comparable overall leukocyte frequencies (Additional file 1: Figures S10, S11). Thus, while SFL populations are not fully reflective of the synovium environment, the SF is a more readily available sample for understanding the immunobiology of the joint.

Some of the limitations of our study include the exclusive use of a patient population that was in enough discomfort to seek medical treatments, thus having a limited number of patients in early stages of the disease (KL grade II, *N* = 20) and very few at even earlier stages (KL grade I, *N* = 4); future studies could focus on these earlier populations in which the MΦ subset ratio effects may be more pronounced. An additional limitation of our study is the fact that our samples were acquired from only one hospital, leading to a preselection of patients referred to it; future studies would require the use of multicentered cohorts. Patient samples were acquired from those presenting SF, excluding a subset of the KOA population with low volumes of SF, unavailable for harvest. Last, since our study was designed to determine the frequencies of SFLs and not their numbers, we were not able to simultaneously quantify absolute numbers of leukocytes, which could have allowed us to uncover other possible predictors of PROMs.

## Conclusions

SF MΦ subsets correlate with KOA stiffness, function and quality of life. These correlations are supportive of a biological role of MΦ subsets in KOA inflammation, which warrants further study. SF MΦs may also be investigated both as biomarkers of symptomatic KOA and as targets of novel KOA therapeutics.

## Additional file

Additional file 1: Supplementary Methods. **Figure S1.** Gating method for determining monocyte/macrophage frequencies and subpopulations ratios. **Table S1.** Descriptive statistics of patients with knee osteoarthritis (KOA) from whom synovial fluid (SF) was acquired. **Table S2.** Pain medications used. **Figure S2.** CD14^+^CD16^+^-monocyte/macrophages (MΦs)/total SF MΦs ratio vs. the use of pain medications. **Figure S3.** Inflammatory MΦ subsets phenotype in KOA SFs and PBMCs. **Figure S4.** CD14+CD16+ MΦs/total SF MΦs at early and late stage radiographic KOA. **Table S3.** Effect size of ratio of MΦ subsets in total SF MΦs on patient reported outcome measures (PROMs) from early KOA (KL I/II) subcohort. **Table S4.** Effect size of ratio of MΦ subsets in total SF MΦs on PROMs from late KOA (KL III/IV) subcohort. **Figure S5.** KOA SF sCD14 and sCD163 correlation. **Table S5.** Effect size of SF sCD14 and sCD163 on PROMs. **Figure S6.** Pearson correlations between SF sCD14 vs PROMS. **Figure S7.** MΦs and the pro-inflammatory MΦs correlate with SF sCD14 levels. **Figure S8.** Positive correlation between SF sCD14 and CD4+ T cell frequency in SF leukocytes (SFLs). **Table S6.** Pearson correlations between MΦs and its subsets vs SF CCL2. **Table S7.** Effect size of SF CCL2 on PROMs. **Figure S9.** Correlation between SF CD4+ T cells and SF MΦs. **Figure S10.** Synovium leukocyte populations and SFLs. **Figure S11.** Synovium leukocyte and SFL MΦ subsets. **Figure S12.** Pearson correlations between CD14^+^CD16^+^-MΦs/total SF MΦs vs. PROMs. (PDF 2642 kb)

## Abbreviations

ADL: Activity of daily living
BMI: Body mass index
CCL2: Chemokine (C-C motif) ligand 2
CI: Confidence intervals
CX3CL1: Chemokine (C-X3-C motif) ligand 1
HLA-DR: Human leukocyte antigen–antigen D related
KL: Kellgren–Lawrence
KOA: Knee osteoarthritis
KOOS: Knee Injury and Osteoarthritis Injury Score
MΦ: Monocytes/macrophages
OA: Osteoarthritis
PBMCs: Peripheral blood mononuclear cells
PROMs: Patient-reported outcome measures
QOL: Quality of life
RA: Rheumatoid arthritis
SF: Synovial fluid
SFLs: Synovial fluid leukocytes
WOMAC: The Western Ontario and McMaster Universities Osteoarthritis Index
