# Supplementary material for: Synovial fluid monocyte/macrophage subsets and their correlation to patient-reported outcomes in osteoarthritic patients: a cohort study
Source: Arthritis Res Ther. 2019 Jan 18;21:26. doi: 10.1186/s13075-018-1798-2 (PMC6339358; doi:10.1186/s13075-018-1798-2)
Supplement: Supplementary file 1 — Supplementary Methods. Figure S1. Gating method for determining monocyte/macrophage frequencies and subpopulations ratios. Table S1. Descriptive statistics of patients with knee osteoarthritis (KOA) from whom synovial fluid (SF) was acquired. Table S2. Pain medications used. Figure S2. CD14+CD16+-monocyte/macrophages (MΦs)/total SF MΦs ratio vs. the use of pain medications. Figure S3. Inflammatory MΦ subsets phenotype in KOA SFs and PBMCs. Figure S4. CD14+CD16+ MΦs/total SF MΦs at early and late stage radiographic KOA. Table S3. Effect size of ratio of MΦ subsets in total SF MΦs on patient reported outcome measures (PROMs) from early KOA (KL I/II) subcohort. Table S4. Effect size of ratio of MΦ subsets in total SF MΦs on PROMs from late KOA (KL III/IV) subcohort. Figure S5. KOA SF sCD14 and sCD163 correlation. Table S5. Effect size of SF sCD14 and sCD163 on PROMs. Figure S6. Pearson correlations between SF sCD14 vs PROMS. Figure S7. MΦs and the pro-inflammatory MΦs correlate with SF sCD14 levels. Figure S8. Positive correlation between SF sCD14 and CD4+ T cell frequency in SF leukocytes (SFLs). Table S6. Pearson correlations between MΦs and its subsets vs SF CCL2. Table S7. Effect size of SF CCL2 on PROMs. Figure S9. Correlation between SF CD4+ T cells and SF MΦs. Figure S10. Synovium leukocyte populations and SFLs. Figure S11. Synovium leukocyte and SFL MΦ subsets. Figure S12. Pearson correlations between CD14+CD16+-MΦs/total SF MΦs vs. PROMs. (PDF 2642 kb) [file 13075_2018_1798_MOESM1_ESM.pdf]

Supplementary Methods

SF and peripheral blood mononuclear cell isolation

SF samples with obvious blood contamination were not included in the study. SFs were centrifuged at 12,000xg for 15 min, followed by removal of SF supernatant, which was stored at -80ºC for subsequent analysis. The cell pellet was resuspended in 100 U/ml Hyaluronidase in RPMI (~1 SF:1 Hyaluronidase solution), left at 37ºC for 15 min to reduce SF viscosity. In pilot experiments, SF supernatants were checked for the presence of cells after 12,000xg centrifugation, and we were unable to find any cells by microscopic examination. Lower centrifugation forces and times did not pellet all cells. Hyaluronidase treatment was performed after first centrifugation, as the pellet remained along with 100-200 ul of SF and needed digestion to reduce cells loss.

Samples were diluted in flow cytometry buffer (phosphate buffered saline, 1mM EDTA and 2% fetal bovine serum), filtered through a 70 µm cell strainer and washed to obtain SF cells.

Peripheral blood mononuclear cells (PBMCs) were isolated by density gradient centrifugation on Lymphoprep (Stem Cell Technologies, BC, Canada) from fresh blood samples as described by manufacturer.

No erythrocyte lysis buffer was used before flow cytometry or any other step of the procedure. Erythrocytes present in any of the samples were excluded by immunolabelling for CD45 before flow cytometry.

Cell immunolabeling

For cell immunolabeling, SF cells or PBMCs were incubated for 20-30 min on ice with the isotype, MΦ or T cell antibody panel (all antibodies used were from Biolegend, unless otherwise specified). The isotype panel had matched isotypes to the antibodies used in the MΦ and T cell panels except CD45, where the samples had an obvious negative control population: Mouse IgG1 kappa (MOPC-21) FITC,

PE, BV510, PerCPCy5.5, PeCy7 (Biolegend); Mouse IgG2a kappa (MOPC-173) PerCPCy5.5, APC-Cy7 (Biolegend); and Mouse IgG1 kappa eFluor 660 (clone P3.6.2.8.1) from Thermofisher. The MΦ panel contained: CD163-FITC (clone GHI/61), CD16-PE (clone 3G8) or BV510, HLA-DR-PerCPCy5.5 (clone L243), CD14-PECy7 (clone 61D3, eBioscience) for all samples. In a subset (n=75 for SFLs, n=53 for PBMCs), where we quantified the ratio of cells to total leukocytes, CD45-PEDazzle or Pacific Blue (clone HI30) was used; quantification of frequencies without the use of CD45 was not feasible due to the debris in KOA SFs. CCR2-APC-Cy7 (n=56 SFLs, n=42 PBMCs; K036C2), and 25F9-eFluor 660 (n=26 SFLs, n=7 PBMCs; ThermoFisher) were added to characterize MΦ subsets.

The T cell panel contained: CD4-FITC (clone OKT-4, eBioscience), CD69-PE (clone FN50), CD3-PerCPCy5.5 (clone SK7) and CD25-PECy7 (clone BC96) for all samples. In a subset, as with the MΦ panel, CD45-PEDazzle or APC were added. In a subcohort (n=32 SFCs, n=21 PBMCs), HLA-DR was added to the panel to determine late activation.

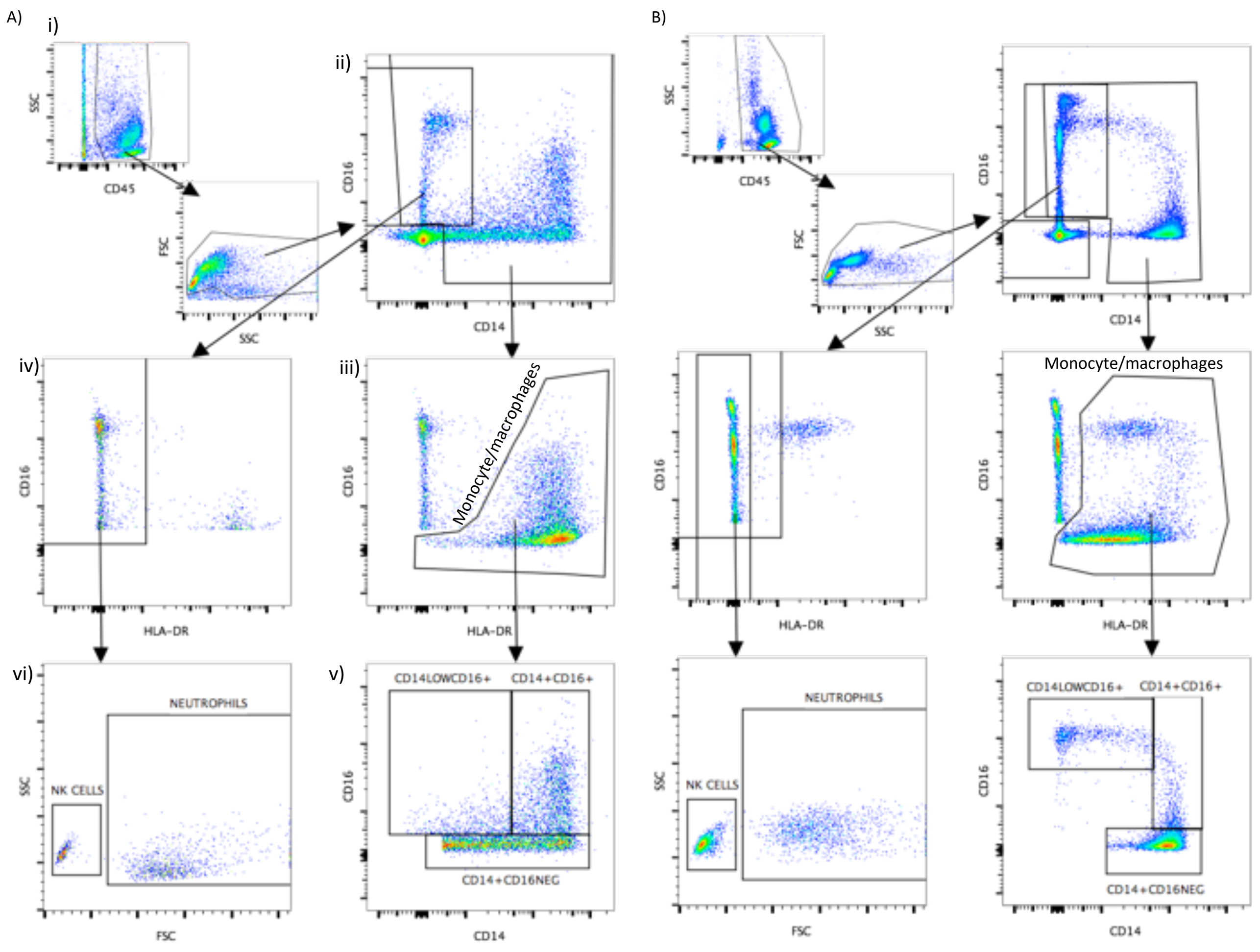

Figure S1: Gating method for determining monocyte/macrophage frequencies and subpopulations ratios. Based on method by *Abeles et al, 2012:* A) Method used on synovial fluid leukocytes (SFLs): i) SFLs were identified from SF cells and particles by their expression of CD45 and FSC/SSC profiles. ii) From SFLs (CD45+), CD16+ and/or CD14+ were selected as possible monocyte/macrophages. CD16+ were selected as possible neutrophils or NK cells. iii)Possible monocyte/macrophages were confirmed to be monocyte/macrophages based on their expression of HLA-DR. iv) While neutrophils and NK cells were identified from the CD16+ populations as those lacking HLA-DR expression. v) Monocyte/macrophages were classified according to their expression of CD16 and CD14. vi) Neutrophils were identified as cells with high granularity while NK cells as those with low granularity. B) Method use on peripheral blood mononuclear cells: Similar to the one used for SFLs, however monocyte/macrophage population distribution differs from that one in OA SFs.

Table S1: Descriptive statistics of KOA patients from whom SF was acquired

Variable	OA patients with PROMS (N=83)
**BMI, median (IQR)**	29.75 (26.98,32.94) kg/m^2
**Age, median (IQR)**	61 (56, 67) years
**Female, n (%)**	50 (60.2%)
**KLI/II (early) vs. KLIII/IV (late), n (%)**	24 (31.6%) vs. 52 (68.4%), N=76
**Knee replacement, n (%)**	41 (49.4%)
**Diabetes, n (%)**	10 (12%)
**KOOS Symptoms, median (range)**	46.4 (7.1-89.3)
**KOOS Pain, median (range)**	44.4 (5.6-91.7)
**KOOS ADL, median (range)**	48.5 (4.4-100)
**KOOS QOL, median (range)**	18.8 (0-87.5)
**KOOS Sports, median (range)**	15 (0-100)
**WOMAC Pain, median (range)**	50 (5-100)
**WOMAC Function, median (range)**	48.5 (4.4-100)
**WOMAC Stiffness, median (range)**	50 (0-100)

IQR: Interquartile range

Table S2: Pain medications used

Medication Use	Never	Sometimes	Daily	Missing data
**Over-the-counter pain medication**	10%	55%	35%	32/83
**NSAIDs**	41%	31%	27%	32/83
**Muscle Relaxants**	80%	18%	2%	32/83
**Narcotics**	74%	14%	12%	33/83
**Antidepressants**	76%	2%	22%	33/83
**Neuroleptics**	88%	4%	8%	32/83

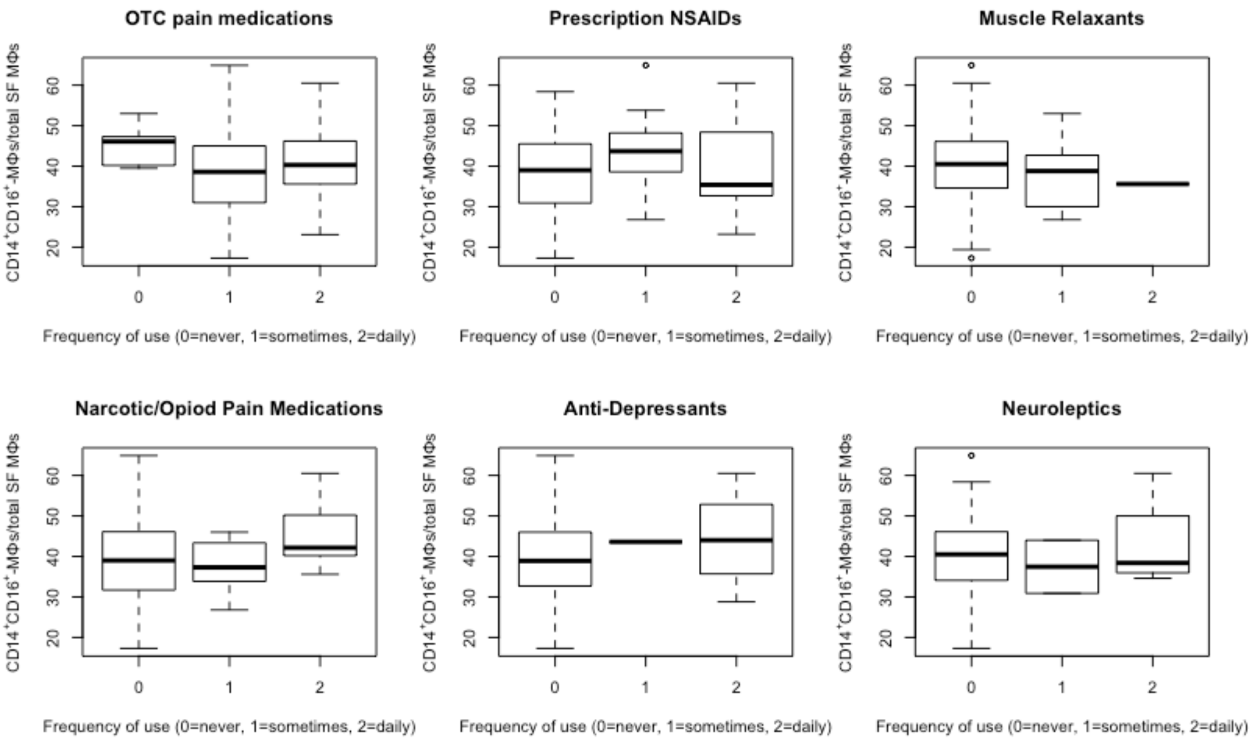

Figure S2: No significant differences in the CD14^{+}CD16^{+}-MΦs/total SF MΦs ratio and the use of pain medications was found using ANOVA. OTC, Over-the-counter; NSAIDs, Non-Steroidal Anti-Inflammatory Drugs; Muscle Relaxants: e.g. Flexeril, Robaxacet, Robaxin; Neuroleptics (Agents to calm nerve pain).

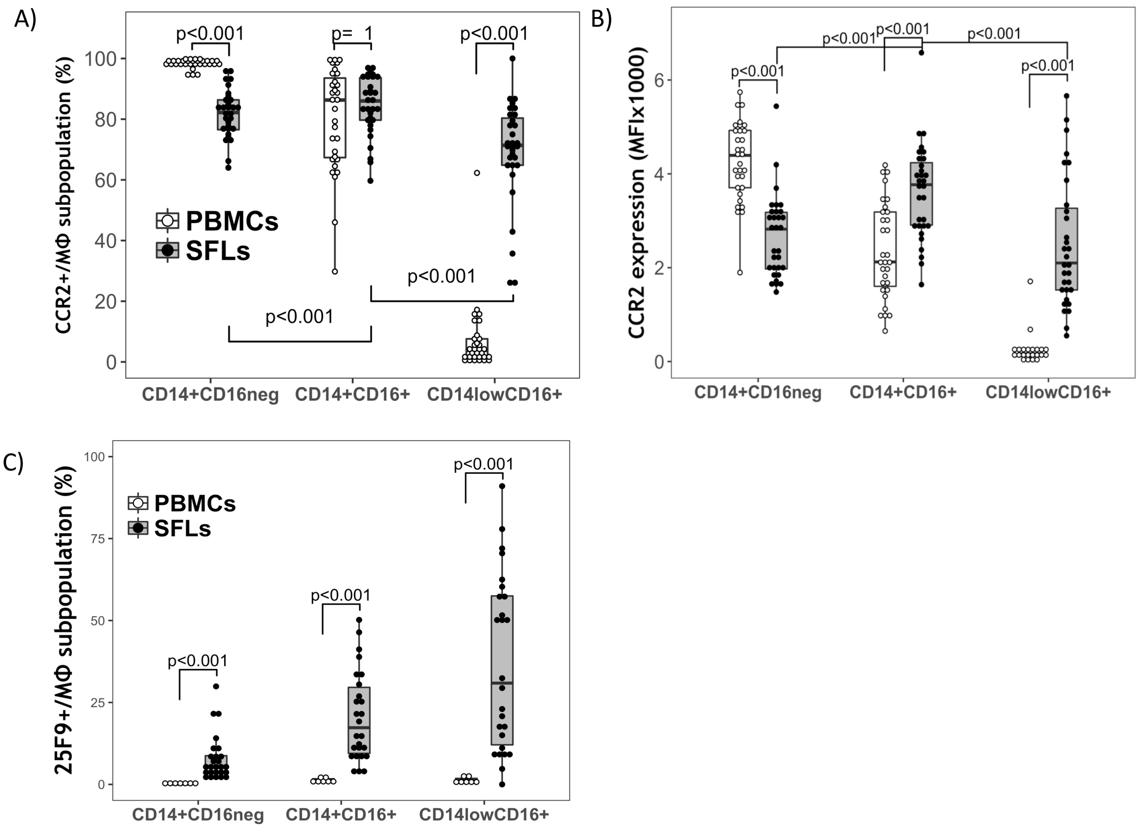

Figure S3: Inflammatory MΦ subsets phenotype in KOA SFs and PBMCs. A) CCR2+ cell frequency and B) CCR2 mean fluorescence intensity (MFI) on PBMCs and SF MΦ subsets (N=31). C) Frequency of MΦs expressing the macrophage marker 25F9 in PBMC and SF MΦs (SFLs, n=26; PBMCs, n=7) (Stat analysis using unpaired tests). Box plots indicate median with interquartile range for box and Tukey-style whiskers. Lines between boxplots indicate significant differences with p value and effects size indicated.

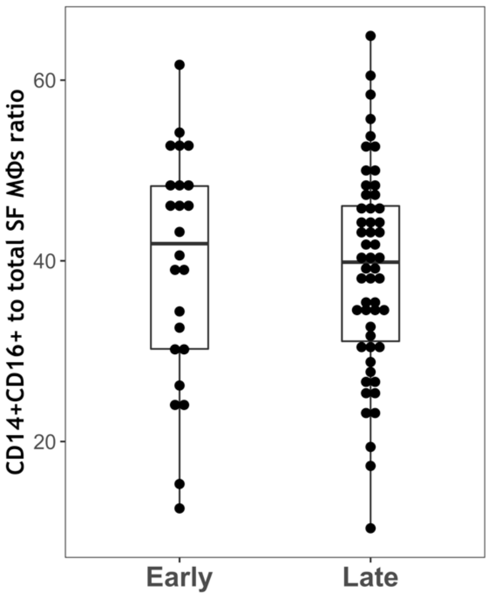

Figure S4: CD14+CD16+ MΦs/total SF MΦs at early (n=26) and late (n=58) stage radiographic KOA. No significant differences found between groups.

Table S3: Effect size of ratio of MΦ subsets in total SF MΦs on patient reported outcome measures (PROMs) from early KOA (KL I/II) subcohort

		**SF CD14+CD16neg-MΦs ratio (%)**	**SF CD14+CD16+-MΦs ratio (%)**	**SF CD14lowCD16+-MΦs ratio (%)**
		**β (95% CI), adjusted p value**	**β (95% CI), adjusted p value**	**β (95% CI), adjusted p value**
Overall				
	mean KOOS	**0.578 (0.285, 1.105), p=0.025**	**-0.691 (-1.287, -0.328), p=0.017**	-0.814 (-3.723, 0.544), p=0.810
	mean WOMAC	**0.583 (0.200, 1.199), p=0.035**	**-0.704 (-1.393, -0.244), p=0.019**	-0.789 (-4.154, 0.508), p=0.832
	SYMPTOMS	0.384 (-0.024, 0.939), p=0.323	-0.467 (-1.065, -0.020), p=0.203	-0.677 (-3.424, 0.940), p=0.978
KOOS	PAIN	**0.494 (0.119, 1.027), p=0.047**	**-0.591 (-1.256, -0.127), p=0.042**	-0.595 (-3.513, 0.913), p=0.895
	ADL	0.639 (0.270, 1.274), p=0.052	**-0.759 (-1.472, -0.272), p=0.038**	-0.929 (-4.222, 0.593), p=0.809
	QOL	**0.684 (0.234, 1.141), p=0.029**	**-0.893 (-1.410, -0.408), p=0.013**	-0.487 (-3.983, 1.173), p=0.997
	SPORTS	**0.690 (0.202, 1.408), p=0.222**	-0.746 (-1.633, -0.166), p=0.226	-1.382 (-5.208, 0.393), p=0.633
	PAIN	0.555 (0.082, 1.220), p=0.083	-0.695 (-1.530, -0.118), p=0.055	-0.591 (-4.007, 0.977), p=0.938
	FUNCTION	0.639 (0.270, 1.274), p=0.052	**-0.760 (-1.470, -0.272), p=0.038**	-0.929 (-4.223, 0.595), p=0.810
	STIFFNESS	0.554 (0.052, 1.264), p=0.182	-0.657 (-1.441, -0.125), p=0.102	-0.848 (-4.284, 0.986), p=0.943

All correlations adjusted for sex, BMI and age; N=24.
Bolded=significant effect estimates (β). Synovial fluid leukocytes, SFLs.

Table S4: Effect size of ratio of MΦ subsets in total SF MΦs on patient reported outcome measures (PROMs) from late KOA (KL III/IV) subcohort

		SF CD14+CD16neg-MΦs ratio (%)	SF CD14+CD16+-MΦs ratio (%)	SF CD14lowCD16+-MΦs ratio (%)
		**β (95% CI), adjusted p value**	**β (95% CI), adjusted p value**	**β (95% CI), adjusted p value**
Overall				
	mean KOOS	0.256 (0.015, 0.519), p=0.282	-0.301 (-0.605, -0.005), p=0.292	-0.361 (-1.139, 0.511), p=0.921
	mean WOMAC	0.321 (-0.039, 0.680), p=0.404	-0.369 (-0.779, 0.072), p=0.415	-0.485 (-1.503, 0.574), p=0.907
	SYMPTOMS	0.038 (-0.236, 0.381), p=0.999	-0.117 (-0.526, 0.192), p=0.962	0.276 (-0.524, 1.148), p=0.968
KOOS	PAIN	0.181 (-0.200, 0.531), p=0.841	-0.200 (-0.619, 0.256), p=0.871	-0.371 (-1.328, 0.679), p=0.969
	ADL	0.395 (0.018, 0.794), p=0.275	-0.375 (-0.837, 0.123), p=0.507	-0.996 (-2.163, 0.189), p=0.306
	QOL	0.296 (0.014, 0.506), p=0.181	**-0.425 (-0.649, -0.159), p=0.037**	0.100 (-0.785, 1.065), p=1.000
	SPORTS	0.370 (0.047, 0.858), p=0.272	-0.387 (-1.008, 0.036), p=0.475	-0.814 (-1.815, 0.099), p=0.268
	PAIN	0.185 (-0.171, 0.540), p=0.810	-0.192 (-0.614, 0.246), p=0.879	-0.458 (-1.464, 0.650), p=0.939
	FUNCTION	0.394 (0.018, 0.794), p=0.275	-0.375 (-0.837, 0.123), p=0.507	-0.995 (-2.162, 0.190), p=0.306
	STIFFNESS	0.382 (-0.052, 0.787), p=0.386	-0.542 (-0.975, -0.059), p=0.201	-0.003 (-1.145, 1.078), p=1.000

All correlations adjusted for sex, BMI and age; N=52.
Bolded=significant effect estimates (β). Synovial fluid leukocytes, SFLs.

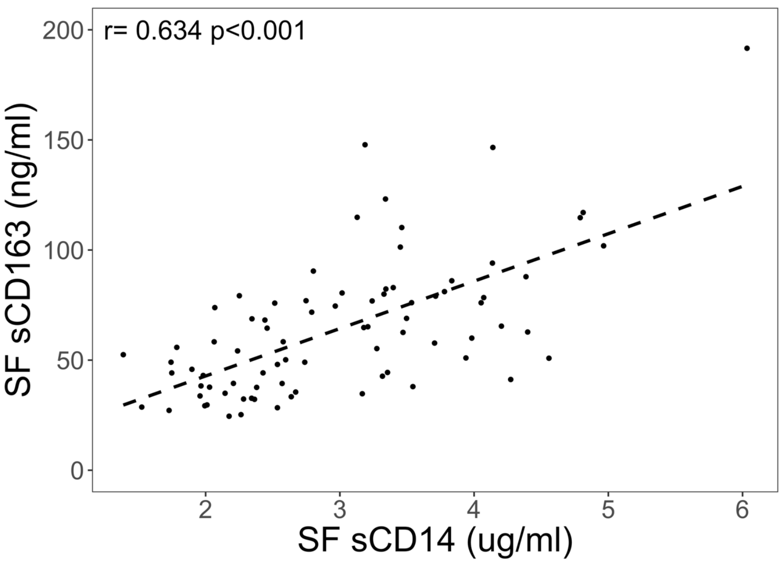

Figure S5: KOA SF sCD14 and sCD163 (N=81) correlation.

Table S5: Effect size of SF sCD14 and sCD163 on patient reported outcome measures (PROMs)

		SF sCD14 (ug/ml)	SF sCD163 (ng/ml)
		**β (95% CI), adjusted p value**	**β (95% CI), adjusted p value**
**Overall scores**			
	**mean KOOS**	-3.56 (-7.69, 0.67), p=0.322	0.00 (-0.14, 0.12), p=1.000
	**mean KOOS@**	-1.84 (-5.70, 2.72), p=0.858	0.00 (-0.12, 0.13), p=1.000
	**mean WOMAC**	-3.39 (-7.80, 0.86), p=0.408	-0.02 (-0.15, 0.09), p=0.993
	**mean WOMAC@**	-1.55 (-5.69, 3.17), p=0.924	-0.02 (-0.13, 0.11), p=0.997
**KOOS**			
	**SYMPTOMS**	-3.25 (-6.95, 0.86), p=0.365	-0.01 (-0.12, 0.12), p=1.000
	**SYMPTOMS@**	-1.63 (-4.98, 2.65), p=0.863	-0.02 (-0.12, 0.10), p=0.986
	**PAIN**	-2.83 (-6.82, 1.40), p=0.541	0.00 (-0.12, 0.11), p=1.000
	**PAIN@**	-1.18 (-5.31, 3.34), p=0.967	0.00 (-0.11, 0.12), p=1.000
	**ADL**	-5.52 (-10.90, -0.48), p=0.127	-0.07 (-0.23, 0.07), p=0.829
	**ADL@**	-3.83 (-9.09, 1.45), p=0.487	-0.07 (-0.21, 0.07), p=0.767
	**QOL**	-4.40 (-8.73, -0.41), p=0.115	0.02 (-0.12, 0.18), p=0.996
	**QOL@**	-2.76 (-6.85, 1.55), p=0.586	0.02 (-0.11, 0.16), p=0.997
	**SPORTS**	-1.84 (-8.57, 7.54), p=0.989	0.06 (-0.18, 0.36), p=0.985
	**SPORTS@**	0.18 (-6.86, 9.72), p=1.000	0.07 (-0.18, 0.39), p=0.982
**WOMAC**			
	**PAIN**	-1.42 (-5.29, 2.77), p=0.925	0.00 (-0.11, 0.12), p=1.000
	**PAIN@**	0.29 (-3.48, 5.03), p=1.000	0.02 (-0.09, 0.15), p=0.998
	**FUNCTION**	-5.52 (-10.90, -0.48), p=0.127	-0.07 (-0.23, 0.07), p=0.829
	**FUNCTION@**	-3.83 (-9.09, 1.45), p=0.486	-0.07 (-0.21, 0.07), p=0.767
	**STIFFNESS**	-3.25 (-8.19, 1.62), p=0.547	-0.01 (-0.14, 0.12), p=1.000
	**STIFFNESS@**	-1.09 (-5.93, 3.99), p=0.985	0.01 (-0.12, 0.13), p=1.000

All correlations adjusted for sex, BMI and age; N=79.
Bolded=significant effect estimates (β). Synovial fluid leukocytes, SFLs.
@Additionally adjusted for OA state (i.e. early:KLI/II, n=23; or late:KLIII/IV, n=50); N=73

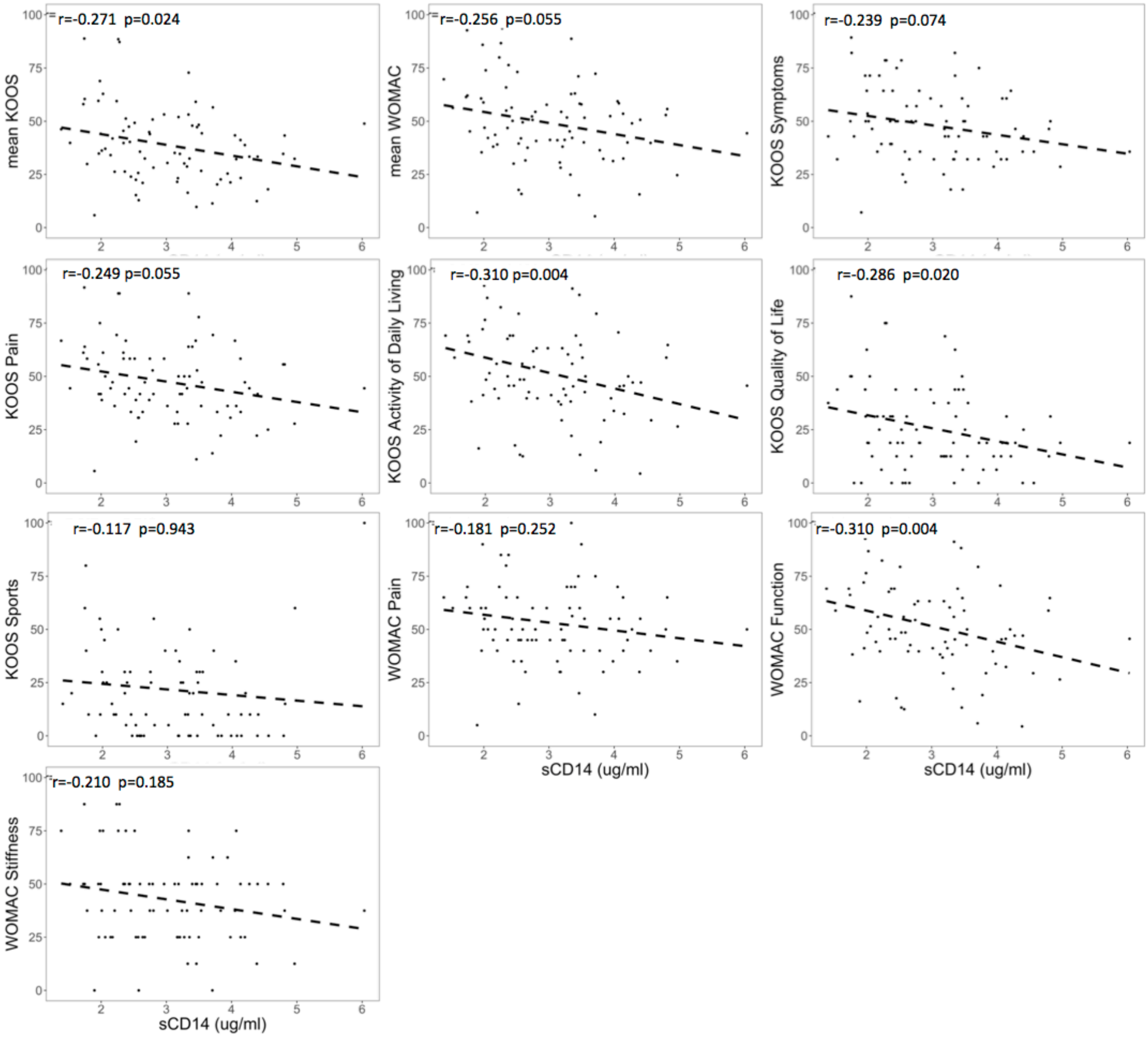

Figure S6: Pearson correlations between SF sCD14 (ug/ml) vs PROMS (N=79); p values adjusted for multiple comparisons. No adjustment for covariates.

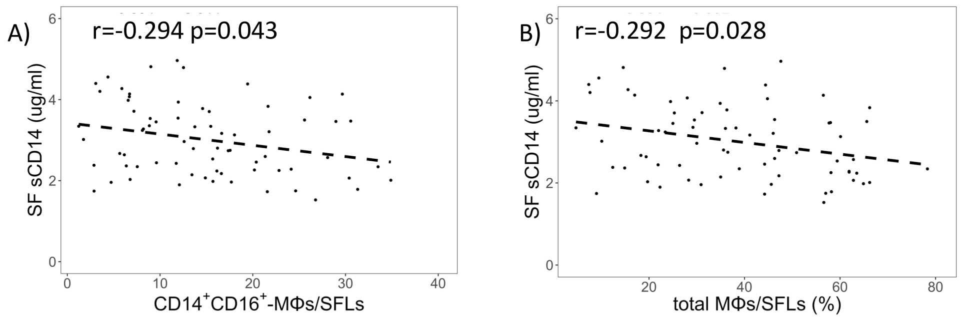

Figure S7: MΦs and the pro-inflammatory MΦs correlate with SF sCD14 levels. A) Negative Correlation between SF sCD14 levels and CD14+CD16+-MΦs frequency in SFLs ratio, N=81. B) Negative Correlation between SF sCD14 levels and total MΦs frequency in SFLs ratio, N=81 Pearson r and adjusted p value. Dashed line indicates regression resulting from general linear modelling approximation.

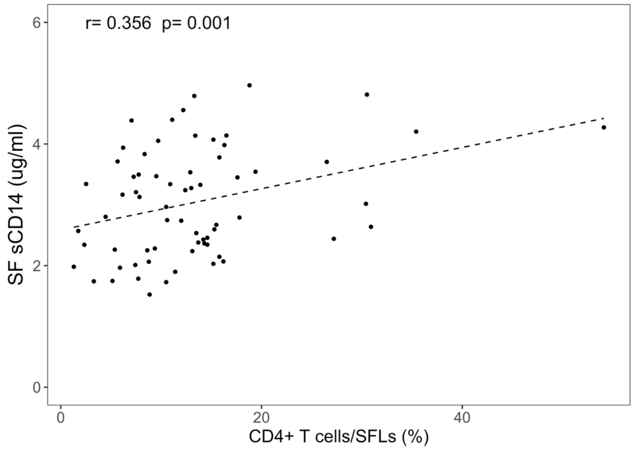

Figure S8: Positive correlation between SF sCD14 and T helper (CD4+) cell frequency in SFLs, N=69

Table S6: Pearson correlations between MΦs and its subsets vs SF CCL2 (ng/ml) (N=81). P values adjusted for multiple comparisons.

	**Pearson's r, CI**	**Adjusted p**
$CD14^{+}CD16^{+}$-MΦs/total SF MΦs	**0.292 (0.113, 0.455)**	**0.004**
$CD14^{+}CD16^{neg}$-MΦs/total SF MΦs	-0.258 (-0.441, -0.042)	0.055
$CD14^{low}CD16^{+}$-MΦs/total SF MΦs	0.079 (-0.166, 0.353)	0.964
total MΦs/total SFLs	0.110 (-0.120, 0.334)	0.817
$CD14^{+}CD16^{+}$-MΦs/total SFLs	0.264 (0.032, 0.467)	0.070
$CD14^{+}CD16^{neg}$-MΦs/total SFLs	-0.038 (-0.238, 0.194)	0.993
$CD14^{low}CD16^{+}$-MΦs/total SFLs	0.124 (-0.156, 0.394)	0.860

*Bolded values indicate significance

Table S7: Effect size of SF CCL2 on patient reported outcome measures (PROMs)

		SF CCL2 (ng/ml)
		**β (95% CI), adjusted p value**
Overall		
	mean KOOS	-83.674 (-230.575, 34.354), p=0.654
	mean KOOS@	-30.730 (-148.157, 63.803), p=0.982
	mean WOMAC	-102.497 (-260.992, 33.620), p=0.564
	mean WOMAC@	-45.954 (-181.149, 62.043), p=0.948
KOOS		
	SYMPTOMS	-83.824 (-210.043, 50.582), p=0.656
	SYMPTOMS@	-40.098 (-160.757, 75.980), p=0.962
	PAIN	-64.263 (-204.610, 55.646), p=0.860
	PAIN@	-16.803 (-139.703, 83.419), p=0.999
	ADL	-104.432 (-293.775, 51.422), p=0.696
	ADL@	-46.034 (-205.696, 82.394), p=0.969
	QOL	-123.178 (-333.174, 26.065), p=0.548
	QOL@	12.795 (-105.539, 115.778), p=1.000
	SPORTS	-0.387 (-1.008, 0.036), p=0.475
	SPORTS@	-63.508 (-255.926, 71.661), p=0.939
WOMAC		
	PAIN	-57.500 (-199.638, 73.180), p=0.924
	PAIN@	-9.782 (-139.085, 104.645), p=1.000
	FUNCTION	-104.407 (-293.652, 51.599), p=0.696
	FUNCTION@	-46.044 (-205.632, 82.392), p=0.969
	STIFFNESS	-145.584 (-310.472, 20.026), p=0.327
	STIFFNESS@	-82.037 (-237.872, 58.905), p=0.788

All correlations adjusted for sex, BMI and age; N=79.
Bolded=significant effect estimates (β). Synovial fluid leukocytes, SFLs.
@Additionally adjusted for OA state (i.e. early:KLI/II, n=23; or late:KLIII/IV, n=50); N=73

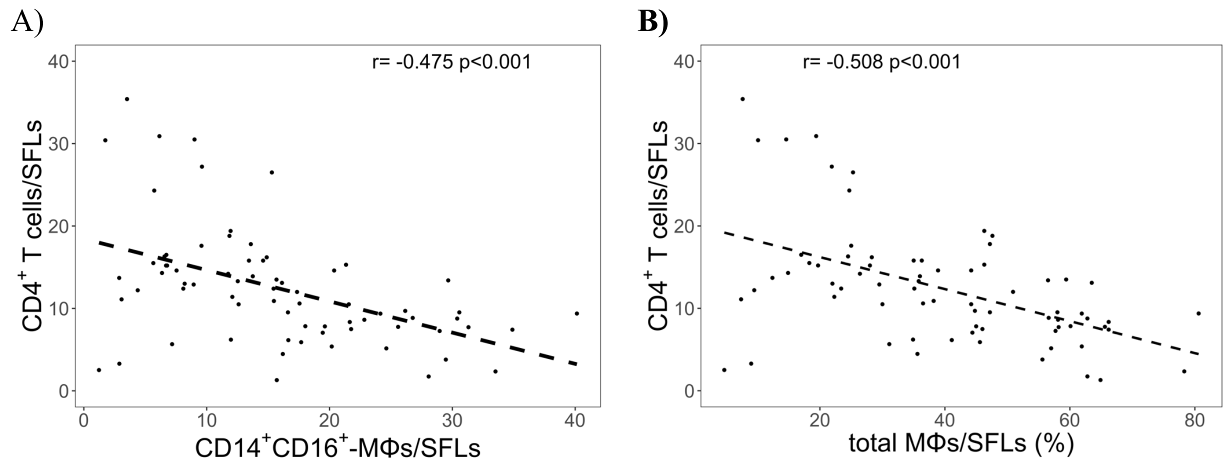

Figure S9: Correlation between SF CD4+ T cells and SF MΦs. A) Inverse correlation between SF CD4$^+$ T cells and CD14+CD16+-MΦs and B) total MΦ (right) frequency in synovial fluid leukocytes (SFLs; N=73)

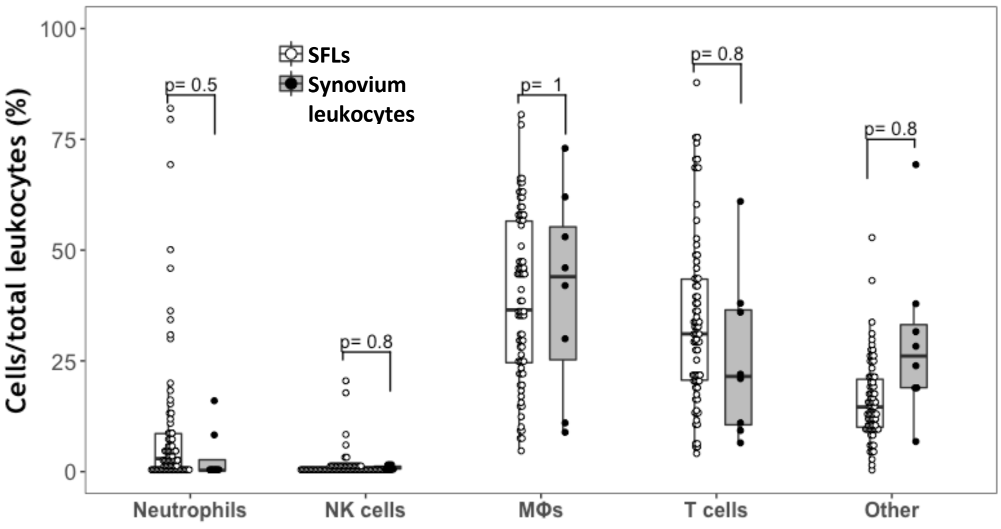

Figure S10: Synovium leukocyte populations and synovial fluid leukocytes (SFLs); n=8 for synovium leukocytes and n=75 SFLs. Unpaired Wilcoxon ran sum test used for comparisons. P values adjusted for multiple comparisons.

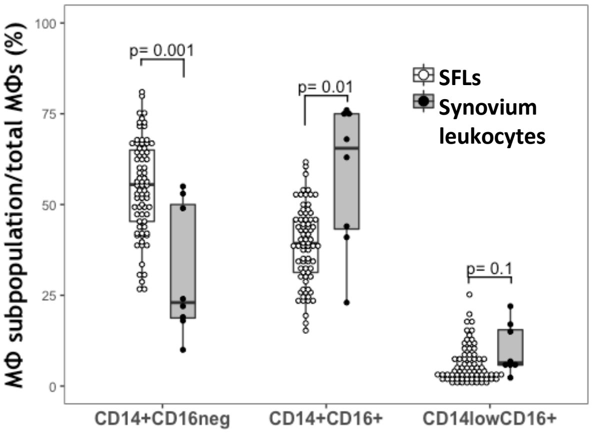

Figure S11: Synovium leukocyte and synovial fluid leukocyte monocyte/macrophage subsets; n=8 for synovium leukocytes and n=73 SFLs. Unpaired Wilcoxon ran sum test used for comparisons. P values adjusted for multiple comparisons.

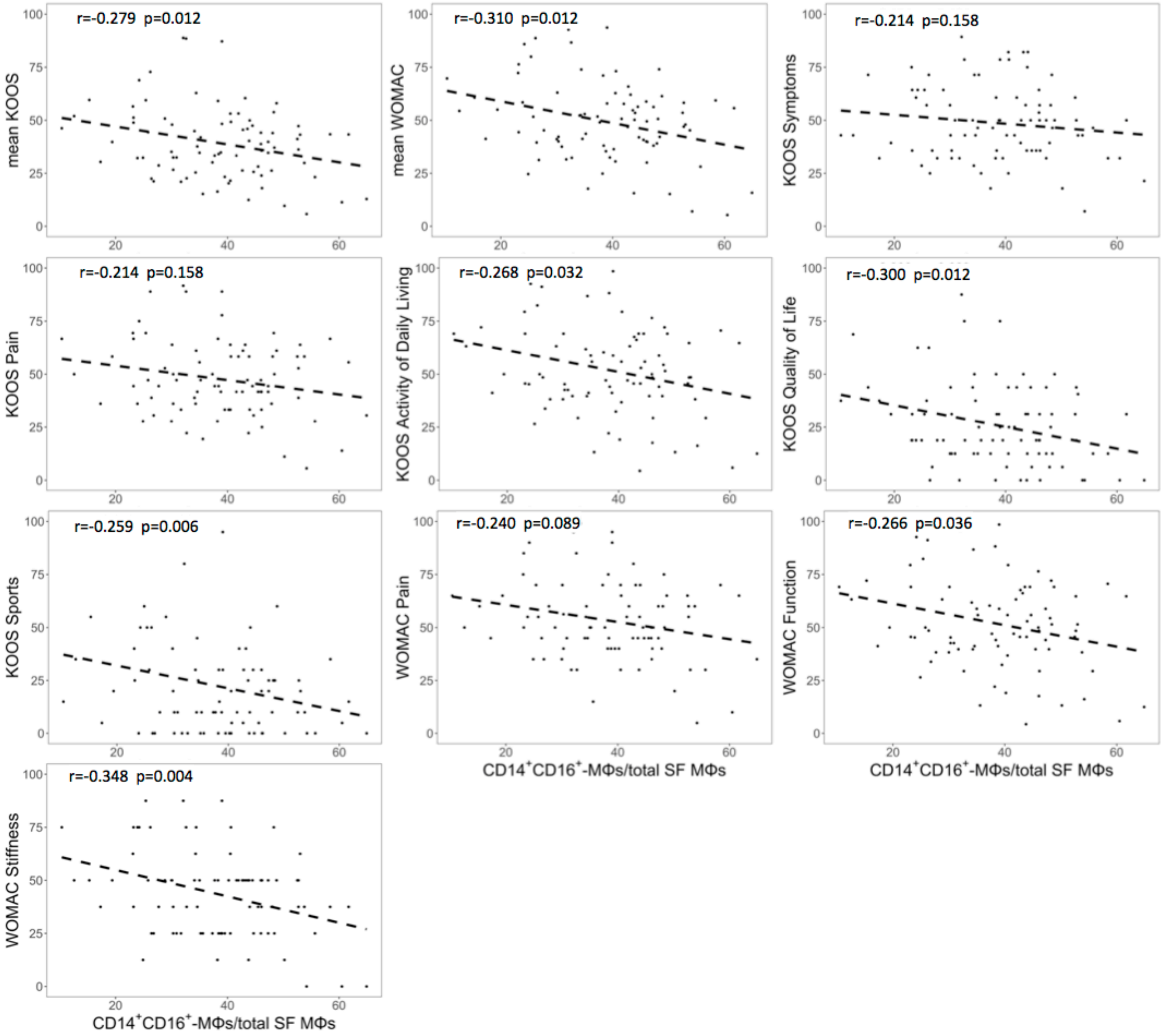

Figure S12: Pearson correlations between CD14$^+$CD16$^+$-MΦs/total SF MΦs vs PROMS (N=83). P values adjusted for multiple comparisons. No adjustment for covariates.
